# Antiretroviral therapy retention, adherence, and clinical outcomes among postpartum women with HIV in Nigeria

**DOI:** 10.1371/journal.pone.0302920

**Published:** 2024-08-07

**Authors:** Clara M. Young, Charlotte A. Chang, Atiene S. Sagay, Godwin Imade, Olabanjo O. Ogunsola, Prosper Okonkwo, Phyllis J. Kanki

**Affiliations:** 1 College of Public Health, The University of Iowa, Iowa City, Iowa, United States of America; 2 Department of Immunology and Infectious Diseases, Harvard T.H. Chan School of Public Health, Boston, Massachusetts, United States of America; 3 Jos University Teaching Hospital, University of Jos, Jos, Nigeria; 4 APIN Public Health Initiatives, Abuja, Nigeria; University of the Witwatersrand, SOUTH AFRICA

## Abstract

While research involving pregnant women with HIV has largely focused on the antepartum and intrapartum periods, few studies in Nigeria have examined the clinical outcomes of these women postpartum. This study aimed to evaluate antiretroviral therapy retention, adherence, and viral suppression among postpartum women in Nigeria. This retrospective clinical data analysis included women with a delivery record at the antenatal HIV clinic at Jos University Teaching Hospital between 2013 and 2017. Descriptive statistics quantified proportions retained, adherent (≥95% medication possession ratio), and virally suppressed up to 24 months postpartum. Among 1535 included women, 1497 met the triple antiretroviral therapy eligibility criteria. At 24 months, 1342 (89.6%) women remained in care, 51 (3.4%) reported transferring, and 104 (7.0%) were lost to follow-up. The proportion of patients with ≥95% medication possession ratio decreased from 79.0% to 69.1% over the 24 months. Viral suppression among those with results was 88.7% at 24 months, but <62% of those retained had viral load results at each time point. In multiple logistic regression, predictors of loss to follow-up included having a more recent HIV diagnosis, higher gravidity, fewer antenatal care visits, and a non-hospital delivery. Predictors of viral non-suppression included poorer adherence, unsuppressed/missing baseline viral load, lower baseline CD4+ T-cell count, and higher gravidity. Loss to follow-up rates were lower and antiretroviral therapy adherence rates similar among postpartum women at our study hospital compared with other sub-Saharan countries. Longer follow-up time and inclusion of multiple facilities for a nationally representative sample would be beneficial in future studies.

## Introduction

Since the human immunodeficiency virus (HIV) epidemic’s peak in 1995, new infections have decreased by 59% to 1.3 million in 2022 [1]. This wane can be widely attributed to the accessibility and evolution of antiretroviral therapy (ART), which has positively shifted patient prognosis and decreased transmission risk. Nonetheless, HIV persists as a leading cause of death in low-income countries–remaining a global health priority [2].

Disproportionately affected by the disease, sub-Saharan Africa constituted 60% of global HIV infections in 2020 [3]. Within the central-west subregion, Nigeria bears the greatest disease burden, ranking fourth in the world [4]. The prioritization of HIV programming in the country has resulted in attenuated incidence and HIV-related morbidity and mortality; however challenges persist [5]. New infections continue to be fueled through mother-to-child transmission (MTCT) during gestation, delivery, and breastfeeding, with 21,000 newly infected children in Nigeria in 2020 [3]. ART coverage among pregnant and breastfeeding women living with HIV in Nigeria was estimated at 44%, with a 25% vertical transmission rate in 2020 [3].

While MTCT research has historically centered on ART uptake and viral suppression among pregnant women living with HIV through delivery and diagnostic outcomes among their neonates after delivery, less coverage has been given to ART continuation and clinical outcomes among these women postpartum [6]. Postnatal continuation of maternal ART can reduce MTCT rates to less than 2% in resource-limited countries [7–9]. However, ART adherence declines dramatically in mothers with HIV up to 18 months postpartum [10], and new mothers have increased risks of becoming lost to follow-up (LTFU) in HIV care and having viremia, increasing risk of transmission to infants [11, 12]. Suboptimal adherence or discontinuation of ART endangers maternal health, even increasing the odds of death [13, 14]; with mothers often the primary familial caregivers, poor maternal health, in turn, endangers their children’s health.

To achieve the United Nations Programme on HIV/AIDS (UNAIDS) 95-95-95 goals for the year 2030—that 95% of those living with HIV know their status, have ART, and be virally suppressed—it is crucial to assess ART retention, adherence, and viral suppression among all key populations, including postnatal women [15, 16]. We retrospectively measured postpartum retention, ART adherence, and viral suppression in an HIV care program in Nigeria. We additionally identified demographic and clinical risk factors for postpartum LTFU and unsuppressed viral load.

## Materials and methods

### Patient population

The study population included 1535 pregnant women living with HIV-1, ≥18 years of age, attending the antenatal HIV clinic at Jos University Teaching Hospital (JUTH), north-central Nigeria, with a recorded delivery between 2013 and 2017. Demographic and clinical data were extracted from the clinical databases at enrollment in the HIV program, ART initiation, antenatal booking, delivery, and up to 24 months after delivery, with data censored on 31 December 2019. The data were collected for routine clinical management as part of the APIN Public Health Initiatives HIV program at JUTH, supported by the President’s Emergency Plan for AIDS Relief (PEPFAR).

Per routine care, patients initiating ART in the adult HIV clinic at JUTH had scheduled medical examinations with CD4+ T-cell counts at baseline and every six months thereafter, and viral load enumeration at 6 months, 12 months, and every 12 months thereafter (with enhanced adherence counseling and additional viral load monitoring if viremic). If a patient with HIV became pregnant or a pregnant woman newly tested HIV-positive, they were enrolled in the antenatal HIV clinic at JUTH. Pregnant women newly initiating treatment received an additional viral load test at initiation. After delivery, they transferred back to the general adult HIV clinic for routine HIV care and returned to the adult monitoring schedule. If a woman receiving antenatal HIV care at JUTH delivered outside of JUTH, their delivery information was recorded when they returned to the clinic with their newborn. All clinical, laboratory, and pharmacy data collected in medical record forms at the adult and antenatal HIV clinics were routinely entered into electronic databases.

ART eligibility conformed to the Nigerian National Guidelines for HIV Prevention and Care, which were revised over the study years [17–19]. Between 2013 and 2014, all adults with WHO clinical stage III or IV or CD4+ T-cell counts less than 350 cells/mm^3^ were triple ART-eligible. Ineligible pregnant women received zidovudine and lamivudine during pregnancy with single-dose nevirapine at delivery as prophylaxis. In 2014, Nigerian ART eligibility guidelines were revised to include adults with CD4+ T-cell counts less than 500 cells/mm^3^; to allow for program implementation delays, we considered women meeting these criteria ART-eligible starting 1 January 2015. In 2016, Nigeria expanded eligibility to all pregnant women with HIV per the WHO Option B+ guideline; to allow time for implementation, we considered all women ART-eligible by 1 January 2017.

This project was approved by the APIN Public Health Initiatives and Harvard T.H. Chan School of Public Health Institutional Review Boards. Data used were from patients who provided written informed consent for the use of their data for secondary research at the time of enrollment in the HIV program. The clinical records, which included identifiable information, were accessed on June 17, 2020 by the data manager, who assembled the dataset and removed all identifiers before statistical analyses.

### Measurement and definitions of outcomes

#### ART adherence

ART adherence was measured using pharmacy refill data [20]. Medication possession ratios (MPR) were calculated as the percentage of all days supplied with ART over the total days in each 6-month postpartum time interval, and were categorized as <80%, 80%-94.9%, and ≥95%. New patients picked up ART monthly during their first year; if adherent and virally suppressed, they could pick up a two-month supply bimonthly. Patients were excluded from the adherence analysis if they were receiving ART prophylactically. Patients who became LTFU during the study period were included in the analysis up to the time interval during which their last clinic visit was recorded.

#### Loss to follow-up

While LTFU definitions vary by program and over the years, overlapping with current definitions of interruption in treatment, we defined LTFU conservatively as a sustained absence of ≥180 days since the last clinical and pharmacy visits, assessed at 24 months postpartum [21]. Patients who were ART-ineligible or reported transferring to alternate clinics during the analyzed period were removed from evaluation of this outcome.

#### Unsuppressed viral load

Following program cut-offs, an unsuppressed viral load was defined as a viral load measurement of ≥1000 viral copies/mL. Since laboratory tests were not always performed precisely at the study time points, we used the viral load results within the following timeframes that were closest to baseline, month 12, and month 24: 6 months before delivery to 15 days postpartum, 6–18 months postpartum, and 18–30 months postpartum, respectively.

### Independent variables

Baseline age, HIV clinical (prior years of ART, years since HIV diagnosis, delivery year, ART regimen, viral load, CD4+ T-cell count, and ART adherence between 0–6 months postpartum), and antenatal (surviving children, gestational age at antenatal booking, total antenatal visits, delivery site, delivery year, delivery type, term delivery, infant birthweight, and infant feeding method) factors were evaluated for associations with outcomes, with baseline defined as at or closest to the time of delivery. Other demographic information (marital status, education, and occupation) and previous ART experience were only collected in the ART enrollment record, which was completed whenever the patient initiated ART in the PEPFAR program.

### Statistical analysis

Continuous variables were converted to ordinal by interquartile range (IQR) and clinical categories. Retention, ART adherence, and viral suppression were measured using simple descriptive statistics. Bivariate analyses were performed to identify potential associations between the independent variables and the following outcomes: LTFU and unsuppressed viral load. Exposure variables with a chi-square p-value <0.2 (or Fisher’s exact for frequencies ≤5) were evaluated in a multiple logistic regression model and retained in the final model if p ≤0.05, after backwards elimination. To account for significant missing viral load data, missing baseline viral load results were coded as a separate category, and a sensitivity analysis was performed to compare those with versus those missing postpartum viral load results using bivariate analyses and multiple logistic regression as above. Analysis was conducted with SAS Studio 2020.1.2.

## Results

### Patient population characteristics

1535 women with delivery records at the JUTH antenatal HIV clinic were included in the analyses (Table 1). The median age at delivery was 33 years (IQR: 29–36). The majority of women were married (66.8%), achieved primary or secondary education (61.7%), held non-income generating occupations (46.2%), and lacked previous ART experience (88.7%) at the time of ART enrollment in the PEPFAR HIV program. Most women were ART-eligible (90.6%) at the time of delivery. The median time since HIV diagnosis was 6.3 years (IQR: 3.3–8.4) and the median duration on ART was 5.6 years (IQR:2.9–8.1). Most patients (91.1%) were receiving an ART regimen without a protease inhibitor at delivery. At delivery, the majority of patients (87.2%) had ≥200 CD4+ T-cells/mm^3^ and 51.9% of patients were virally suppressed, with 38.6% of patients missing a viral load result.

**Table 1:** Baseline characteristics of study population.

Demographic		Number	%
Age at Delivery	Missing Data	1	.
	≤29 years	395	25.75
	30–33 years	458	29.86
	34–36 years	326	21.25
	≥37 years	355	23.14
Marital Status^a^	Missing Data	39	.
	Single/Separated/Divorced	496	33.16
	Married	1000	66.84
Education Status^a^	Missing Data	42	.
	No Formal	130	8.71
	Primary/Secondary	921	61.69
	Tertiary	442	29.6
Occupation Status^a^	Missing Data	45	.
	Non-income Generating	688	46.17
	Professional/Manager	356	23.89
	Labor/Service	446	29.93
**Clinical HIV/ART** ^b^		**Number**	**%**
Previous ART Experience^a^	Missing Data	39	.
	ART Naive	1327	88.7
	ART Experienced	169	11.3
Time since HIV Diagnosis	Missing Data	55	.
	Diagnosis during Pregnancy/Delivery	144	9.73
	≤3 years Prepartum	198	13.38
	3.1–6 years Prepartum	367	24.8
	6.1–8 years Prepartum	337	22.77
	>8 years Prepartum	434	29.32
Duration on ART prior to Delivery	Missing Data	95	.
	<4 years	487	33.82
	4–8 years	586	40.69
	>8 years	367	25.49
Drug Regimen at Delivery	Missing Data	30	.
	Regimens without a Protease Inhibitor	1371	91.1
	Regimens with a Protease Inhibitor	134	8.9
Viral Load at Delivery	Missing Data	592	38.57
	Suppressed (<200 copies/mL)	706	45.99
	Suppressed (200–999 copies/mL)	91	5.93
	Unsuppressed (≥1000 copies/mL)	146	9.51
CD4 Cell Count at Delivery	Missing Data	147	.
	<200 cells/mm^3^	178	12.82
	200–349 cells/mm^3^	402	28.96
	350–500 cells/mm^3^	421	30.33
	>500 cells/mm^3^	387	27.88
**Antenatal**		**Number**	**%**
Plurality	Missing Data	175	.
	1	1338	98.38
	2–3	22	1.62
Gravidity	Missing Data	95	.
	1	96	6.67
	2–3	523	36.32
	≥4	821	57.01
Previous Live Births	Missing Data	147	.
	0	198	14.27
	1	301	21.69
	≥2	889	64.05
Surviving Children	Missing Data	149	.
	0	239	17.24
	1–2	725	52.31
	>2	422	30.45
Previous Abortion	Missing Data	198	.
	0	797	59.61
	≥1	540	40.39
Trimester at First Antenatal Care Visit	Missing Data	11	.
	1st (≤12 weeks)	113	7.41
	2nd (13–26 weeks)	989	64.9
	3rd (≥27 weeks)	422	27.69
Total Antenatal Care Visits	1–2	329	21.43
	3–4	606	39.48
	>4	600	39.09
**Delivery**		**Number**	**%**
Year of Delivery	2013–2014	745	48.53
	2015–2017	790	51.47
Delivery Site	Missing Data	14	.
	Jos University Teaching Hospital	276	18.15
	Other Clinic/Home/Road	1245	81.85
Delivery Type	Missing Data	108	.
	Vaginal/Assisted	1086	76.1
	Emergency/Elective C-Section	341	23.9
Gestational Age at Delivery	Missing Data	127	.
	Pre-term (<37 weeks)	64	4.55
	Full-term (≥37 weeks)	1344	95.45
Infant Birthweight	Missing Data	132	.
	Low (≤2.5 kg)	375	26.73
	Normal/High (>2.5 kg)	1028	73.27
Infant Feed Method at Delivery	Missing Data	33	.
	Exclusive Breast Feeding	1345	89.55
	Breast Milk Substitute Supplement	157	10.45

^a^Denotes variables collected from the ART enrollment record, which was completed when the patient initiated ART in the APIN PEPFAR program. All other variables collected at antenatal booking or at delivery, as indicated.

^b^ART, antiretroviral therapy.

### ART retention

**Fig 1:**
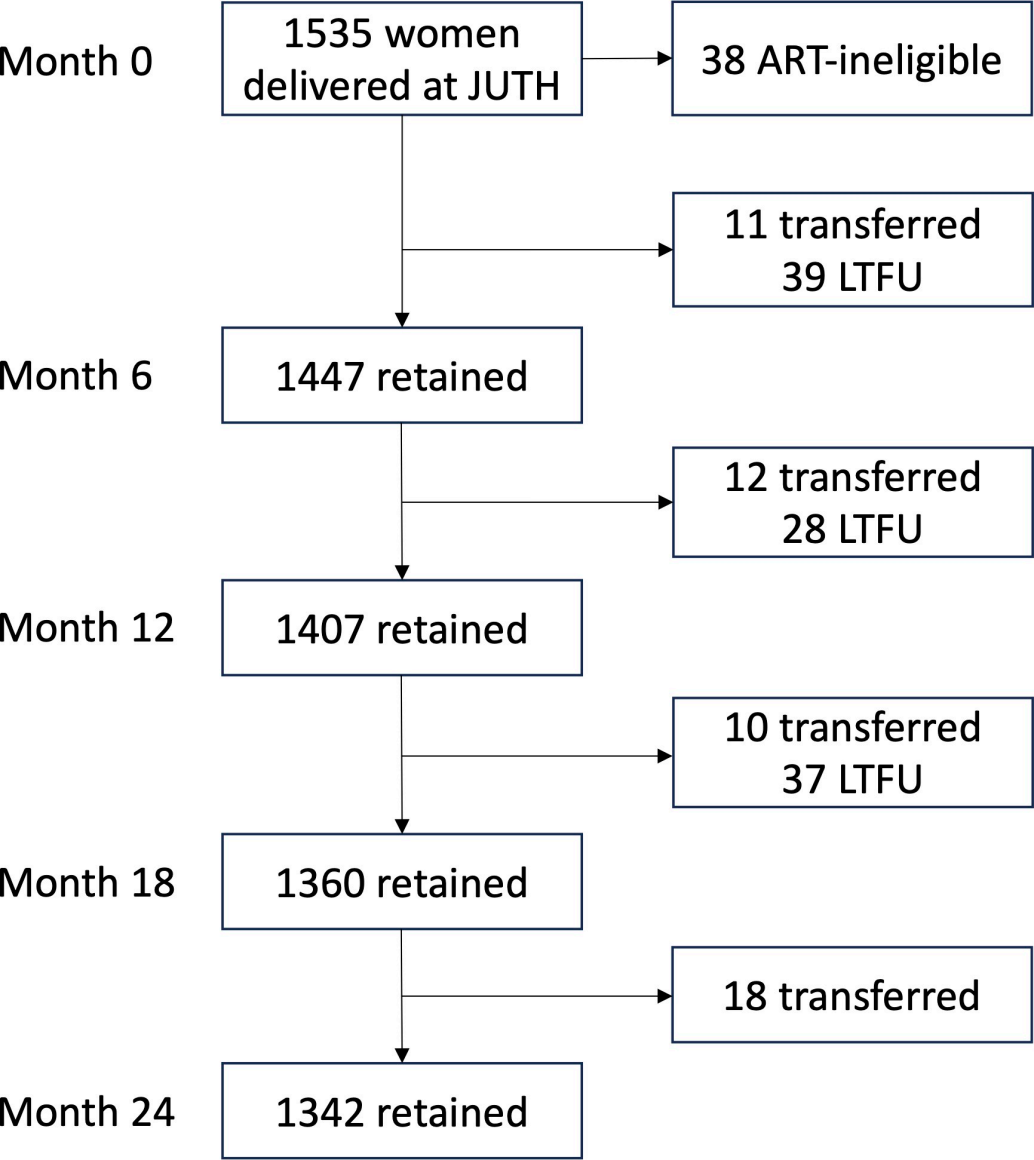
Study population flowchart. Representation of study flow showing numbers of included individuals retained and not retained at each 6-month timepoint. Abbreviations: JUTH, Jos University Teaching Hospital; LTFU, lost to follow-up.

Among 1497 ART-eligible women at delivery, 1342 (89.6%) were retained in care at 24 months postpartum (Fig 1). Cumulatively, 51 (3.4%) women reported transferring to another clinic, and 104 (7.0%) were LTFU.

### ART adherence over time

**Fig 2:**
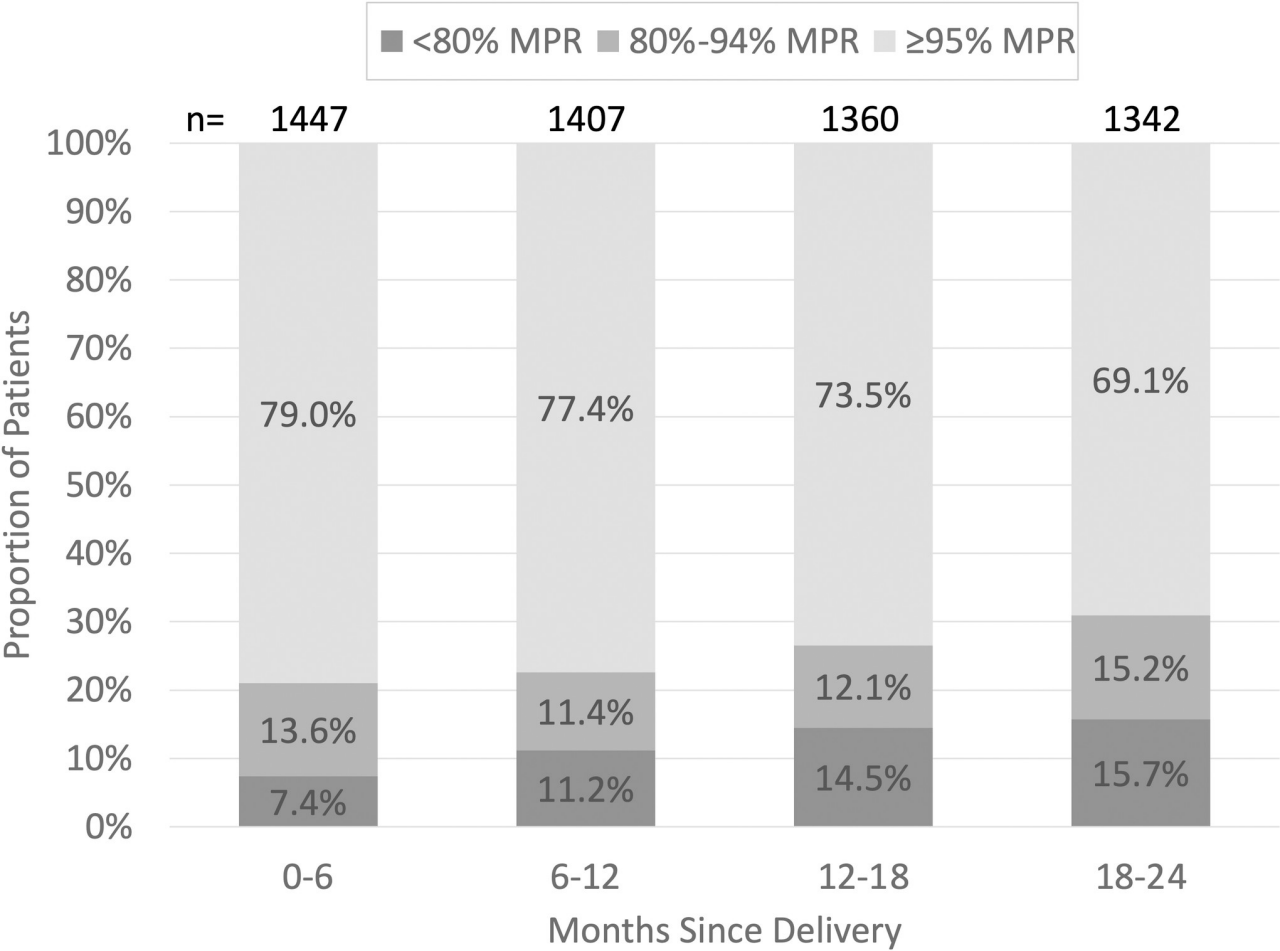
Medication possession ratio. The proportion of patients with <80%, 80%-94.9%, and ≥95% MPR between 0–6, 6–12, 12–18, and 18–24 months after delivery among ART-eligible patients retained in each period. Abbreviations: MPR, medication possession ratio; n, number of patients retained.

Among ART-eligible women retained in each period, mean MPR over time was 95.5% (95% CI 95.0%–96.1%) between 0–6 months, 93.9% (95% CI 93.1%–94.6%) between 6–12 months, 92.0% (95% CI 91.0%–92.9%) between 12–18 months, and 91.0% (95% CI 90.0%–91.9%) between 18–24 months postpartum. The proportion of postpartum patients with ≥95% MPR decreased from 79.0% to 69.1% while the proportion of patients with <80% MPR increased from 7.4% to 15.7% over the study period (Fig 2).

### Viral load suppression

Among all 1497 ART-eligible patients, 926 (61.9%) had a baseline viral load result, 688 (50.0%) had a viral load result at 12 months postpartum, and 858 (57.3%) had a viral load result at 24 months postpartum. Mean viral loads among all recorded results were 26,993 viral copies/mL (95% CI 12,551–41,435) at delivery, 9206 viral copies/mL (95% CI 2107–16,305) at 12 months, and 5796 viral copies/mL (95% CI 3256–8336) at 24 months. Among those with VL results, the proportion of patients with a suppressed viral load was 84.9% (786/926) at delivery, 85.8% (590/688) at 12 months, and 88.7% (761/858) at 24 months.

### Risk factors for LTFU

In chi-square bivariate analysis (Table 2), LTFU was potentially associated with the following variables: maternal age, previous ART experience, years since HIV diagnosis, duration on ART, viral load at delivery, CD4+ T-cell count at delivery, total antenatal care visits, abortion history, gravidity, total surviving children, and delivery site.

**Table 2:** Bivariate analysis of loss to follow up.

	Retained		Loss to Follow Up		Total	chi-square test
	Number	%	Number	%	Number	p-value
**Demographic**						
Age at Delivery						
≤29 years	311	88.6%	40	11.4%	351	0.0044
30–33 years	412	93.4%	29	6.6%	441	
34–36 years	298	95.2%	15	4.8%	313	
≥37 years	320	94.1%	20	5.9%	340	
Marital Status^a^						
Single/Separated/Divorced	441	93.2%	32	6.8%	473	0.6998
Married	873	92.7%	69	7.3%	942	
Education Status^a^						
No Formal	107	90.7%	11	9.3%	118	0.3037
Primary/Secondary	808	92.4%	66	7.6%	874	
Tertiary	397	94.3%	24	5.7%	421	
Occupation Status^a^						
Non-income Generating	607	93.0%	46	7.0%	653	0.3927
Professional/Manager	314	94.3%	19	5.7%	333	
Labor/Service	388	91.7%	35	8.3%	423	
**Clinical HIV/ART** ^**b**^	** **	** **	** **	** **	** **	** **
Previous ART Experience^a^						
ART Naive	1167	92.5%	94	7.5%	1261	0.1856
ART Experienced	147	95.5%	7	4.5%	154	
Time since HIV Diagnosis						
Diagnosis during Pregnancy/Delivery	111	88.1%	15	11.9%	126	0.0215
≤3 years Prepartum	163	90.1%	18	9.9%	181	
3.1–6 years Prepartum	324	92.3%	27	7.7%	351	
6.1–8 years Prepartum	303	93.8%	20	6.2%	323	
>8 years Prepartum	401	95.5%	19	4.5%	420	
Duration on ART prior to Delivery						
<4 years	406	90.0%	45	10.0%	451	0.0052
4–8 years	532	94.0%	34	6.0%	566	
>8 years	340	95.5%	16	4.5%	356	
Drug Regimen at Delivery						
Regimens without a Protease Inhibitor	1226	93.7%	82	6.3%	1308	0.4657
Regimens with a Protease Inhibitor	116	92.1%	10	7.9%	126	
Viral Load at Delivery						
Suppressed (<1000 copies/mL)	724	95.1%	37	4.9%	761	0.0011
Unsuppressed (≥1000 copies/mL)	121	91.7%	11	8.3%	132	
Missing Data	497	89.9%	56	10.1%	553	
CD4 Cell Count at Delivery						
<200 cells/mm^3^	153	89.0%	19	11.0%	172	0.012
200–349 cells/mm^3^	359	93.2%	26	6.8%	385	
350–500 cells/mm^3^	386	95.8%	17	4.2%	403	
>500 cells/mm^3^	342	95.0%	18	5.0%	360	
**Antenatal**						
Plurality						
1	1169	92.7%	92	7.3%	1261	1^c^
2–3	19	95.0%	1	5.0%	20	
Gravidity						
1	83	96.5%	3	3.5%	86	0.0527^c^
2–3	457	94.4%	27	5.6%	484	
≥4	717	91.3%	68	8.7%	785	
Previous Live Births						
0	166	92.2%	14	7.8%	180	0.2881
1	267	95.0%	14	5.0%	281	
≥2	779	92.3%	65	7.7%	844	
Surviving Children						
0	201	93.5%	14	6.5%	215	0.1455
1–2	645	93.9%	42	6.1%	687	
>2	364	90.8%	37	9.2%	401	
Previous Abortion						
0	696	93.9%	45	6.1%	741	0.0764
≥1	473	91.3%	45	8.7%	518	
Trimester at First Antenatal Care Visit						
1st (≤12 weeks)	99	94.3%	6	5.7%	105	0.0705
2nd (13–26 weeks)	872	93.7%	59	6.3%	931	
3rd (≥27 weeks)	360	90.2%	39	9.8%	399	
Total Antenatal Care Visits						
1–2	273	88.3%	36	11.7%	309	< .0001
3–4	529	92.0%	46	8.0%	575	
>4	540	96.1%	22	3.9%	562	
**Delivery**						
Year of Delivery						
2013–2014	637	92.6%	51	7.4%	688	0.7571
2015–2017	705	93.0%	53	7.0%	758	
Delivery Site						
Jos University Teaching Hospital	254	96.6%	9	3.4%	263	0.0122
Other Clinic/Home/Road	1079	92.2%	91	7.8%	1170	
Delivery Type						
Vaginal/Assisted	943	92.1%	81	7.9%	1024	0.0855
Emergency/Elective C-Section	301	95.0%	16	5.0%	317	
Gestational Age at Delivery						
Pre-term (<37 weeks)	57	95.0%	3	5.0%	60	0.7952^c^
Full-term (≥37 weeks)	1174	92.8%	91	7.2%	1265	
Infant Birthweight						
Low (≤2.5 kg)	320	90.9%	32	9.1%	352	0.0791
Normal/High (>2.5 kg)	908	93.7%	61	6.3%	969	
Infant Feed Method at Delivery						
Exclusive Breast Feeding	1180	93.1%	87	6.9%	1267	0.7975
Breast Milk Substitute Supplement	137	92.6%	11	7.4%	148	

^a^Denotes variables collected from the ART enrollment record, which was completed when the patient initiated ART in the APIN PEPFAR program. All other variables collected at antenatal booking or at delivery, as indicated.

^b^ART, antiretroviral therapy.

^c^Fisher’s exact test p-values reported when contingency table observations were less than or equal to five.

**Fig 3:**
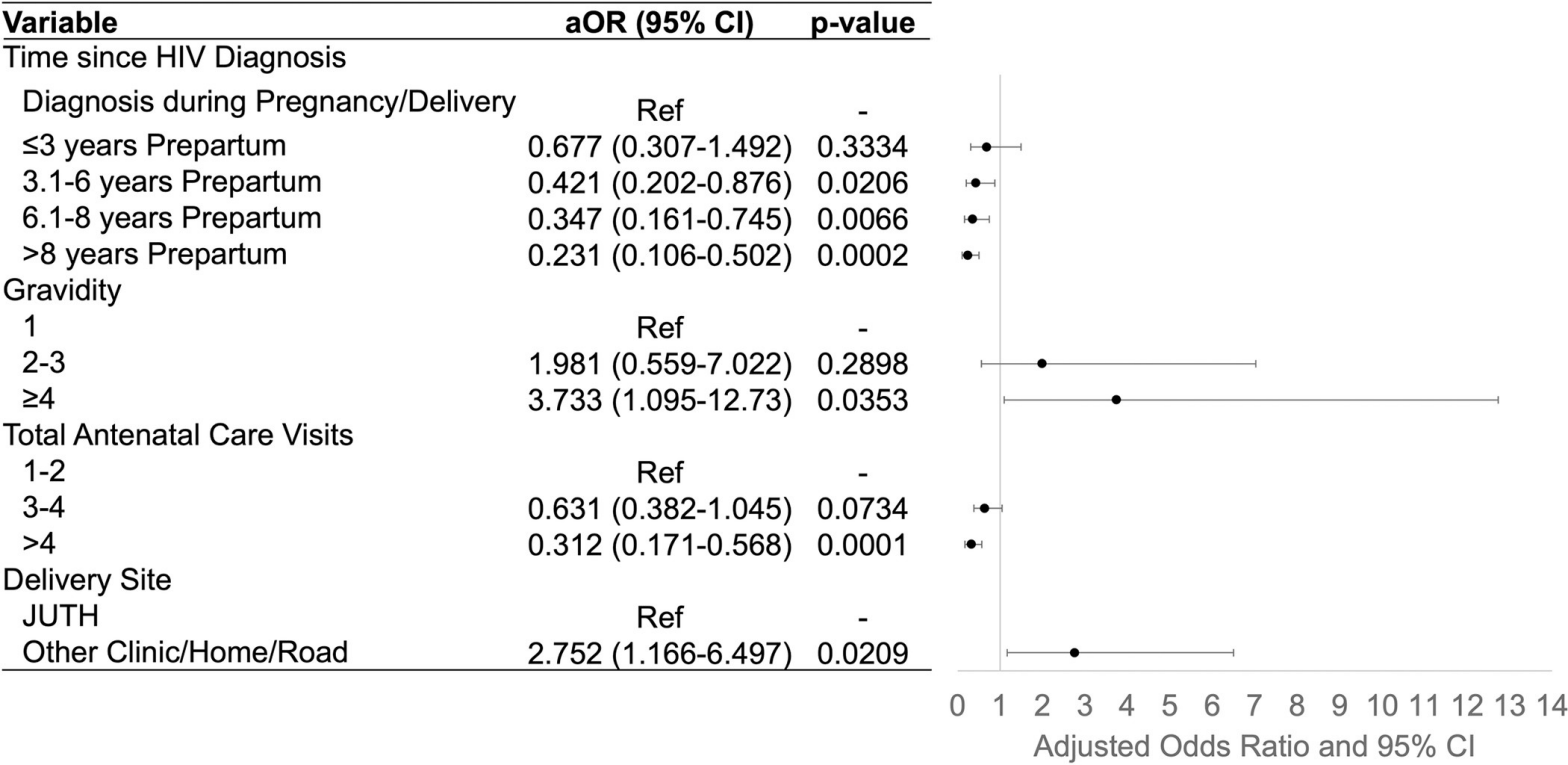
Risk factors for postpartum loss to follow-up. Final multiple logistic regression model shows significant risk factors for women becoming lost to follow-up from the Jos University Teaching Hospital HIV clinic after delivery up to 24 months postpartum. Abbreviations: aOR, adjusted odds ratio; CI, confidence interval; ref, reference group.

1303 patients were retained in the final multiple logistic regression model for risk factors associated with LTFU (Fig 3). A longer time since HIV diagnosis (3.1–6 years, aOR = 0.421, 95% CI 0.202–0.876; 6.1–8 years, aOR = 0.347, 95% CI 0.161–0.745; >8 years, aOR = 0.231, 95% CI 0.106–0.502) and having attended >4 antenatal care visits (aOR = 0.312, 95% CI 0.171–0.568) significantly decreased risk of becoming LTFU. Alternatively, having gravidity of ≥4 pregnancies (aOR = 3.733, 95% CI 1.095–12.73) and delivering outside of JUTH (aOR = 2.752, 95% CI 1.166–6.497) significantly increased risk for becoming LTFU.

### Risk factors for unsuppressed viral load

In chi-square bivariate analysis (Table 3), unsuppressed viral load was potentially associated with the following variables: marital status, education level, occupation, previous ART experience, delivery site, total antenatal care visits, gestational age at antenatal booking, previous live births, surviving children, abortion history, drug regimen, and infant feeding method.

**Table 3:** Bivariate analysis of unsuppressed viral load.

	Suppressed Viral Load		Unsuppressed Viral Load		Total	chi-square test
	Number	%	Number	%	Number	p-value
**Demographic**						
Age at Delivery						
≤29 years	189	85.5%	32	14.5%	221	0.4123
30–33 years	263	83.2%	53	16.8%	316	
34–36 years	182	81.6%	41	18.4%	223	
≥37 years	228	86.7%	35	13.3%	263	
Marital Status^a^						
Single/Separated/Divorced	305	86.9%	46	13.1%	351	0.0663
Married	535	82.4%	114	17.6%	649	
Education Status^a^						
No Formal	50	67.6%	24	32.4%	74	0.0002
Primary/Secondary	522	84.9%	93	15.1%	615	
Tertiary	267	86.4%	42	13.6%	309	
Occupation Status^a^						
Non-income Generating	370	81.5%	84	18.5%	454	0.1074
Professional/Manager	217	87.5%	31	12.5%	248	
Labor/Service	249	84.7%	45	15.3%	294	
**Clinical HIV/ART** ^**b**^						
Previous ART Experience^a^						
ART Naive	749	84.7%	135	15.3%	884	0.0828
ART Experienced	91	78.4%	25	21.6%	116	
Time since HIV Diagnosis						
Diagnosis during Pregnancy/Delivery	61	83.6%	12	16.4%	73	0.3027
≤3 years Prepartum	104	86.7%	16	13.3%	120	
3.1–6 years Prepartum	199	84.7%	36	15.3%	235	
6.1–8 years Prepartum	169	79.7%	43	20.3%	212	
>8 years Prepartum	305	86.2%	49	13.8%	354	
Duration on ART prior to Delivery						
<4 years	243	85.3%	42	14.7%	285	0.2335
4–8 years	310	81.6%	70	18.4%	380	
>8 years	264	86.0%	43	14.0%	307	
Drug Regimen at Delivery						
Regimens without a Protease Inhibitor	788	85.2%	137	14.8%	925	0.0143
Regimens with a Protease Inhibitor	75	75.8%	24	24.2%	99	
Viral Load at Delivery						
Suppressed (<1000 copies/mL)	509	91.7%	46	8.3%	555	< .0001
Unsuppressed (≥1000 copies/mL)	30	39.0%	47	61.0%	77	
Missing Data	324	82.7%	68	17.3%	392	
CD4 Cell Count at Delivery						
<200 cells/mm^3^	64	61.0%	41	39.0%	105	< .0001
200–349 cells/mm^3^	210	80.5%	51	19.5%	261	
350–500 cells/mm^3^	263	88.9%	33	11.1%	296	
>500 cells/mm^3^	248	90.8%	25	9.2%	273	
Adherence 0–6 Months Postpartum						
<95% Medication Possession Ratio	160	76.2%	50	23.8%	210	0.0003
≥95% Medication Possession Ratio	703	86.4%	111	13.6%	814	
**Antenatal**						
Plurality						
1	735	83.0%	151	17.0%	886	0.4878^c^
2–3	14	93.3%	1	6.7%	15	
Gravidity						
1	59	89.4%	7	10.6%	66	0.0751
2–3	295	87.0%	44	13.0%	339	
≥4	450	82.1%	98	17.9%	548	
Previous Live Births						
0	121	90.3%	13	9.7%	134	0.0226
1	172	88.2%	23	11.8%	195	
≥2	486	82.4%	104	17.6%	590	
Surviving Children						
0	133	85.8%	22	14.2%	155	0.0232
1–2	417	87.2%	61	12.8%	478	
>2	227	79.9%	57	20.1%	284	
Previous Abortion						
0	436	82.4%	93	17.6%	529	0.0101
≥1	315	88.7%	40	11.3%	355	
Trimester at First Antenatal Care Visit						
1st (≤12 weeks)	67	80.7%	16	19.3%	83	0.0513
2nd (13–26 weeks)	565	86.7%	87	13.3%	652	
3rd (≥27 weeks)	225	80.9%	53	19.1%	278	
Total Antenatal Care Visits						
1–2	176	80.4%	43	19.6%	219	0.1798
3–4	354	85.9%	58	14.1%	412	
>4	333	84.7%	60	15.3%	393	
**Delivery**						
Year of Delivery						
2013–2014	285	84.1%	54	15.9%	339	0.8984
2015–2017	578	84.4%	107	15.6%	685	
Delivery Site						
Jos University Teaching Hospital	148	80.9%	35	19.1%	183	0.1797
Other Clinic/Home/Road	707	84.9%	126	15.1%	833	
Delivery Type						
Vaginal/Assisted	585	83.7%	114	16.3%	699	0.2351
Emergency/Elective C-Section	206	86.9%	31	13.1%	237	
Gestational Age at Delivery						
Pre-term (<37 weeks)	35	79.5%	9	20.5%	44	0.3832
Full-term (≥37 weeks)	739	84.5%	136	15.5%	875	
Infant Birthweight						
Low (≤2.5 kg)	193	82.8%	40	17.2%	233	0.5975
Normal/High (>2.5 kg)	580	84.3%	108	15.7%	688	
Infant Feed Method at Delivery						
Exclusive Breast Feeding	774	85.3%	133	14.7%	907	0.0069
Breast Milk Substitute Supplement	71	74.7%	24	25.3%	95	

^a^Denotes variables collected from the ART enrollment record, which was completed when the patient initiated ART in the APIN PEPFAR program. All other variables collected at antenatal booking or at delivery, as indicated.

^b^ART, antiretroviral therapy.

^c^Fisher’s exact test p-values reported when contingency table observations were less than or equal to five.

**Fig 4:**
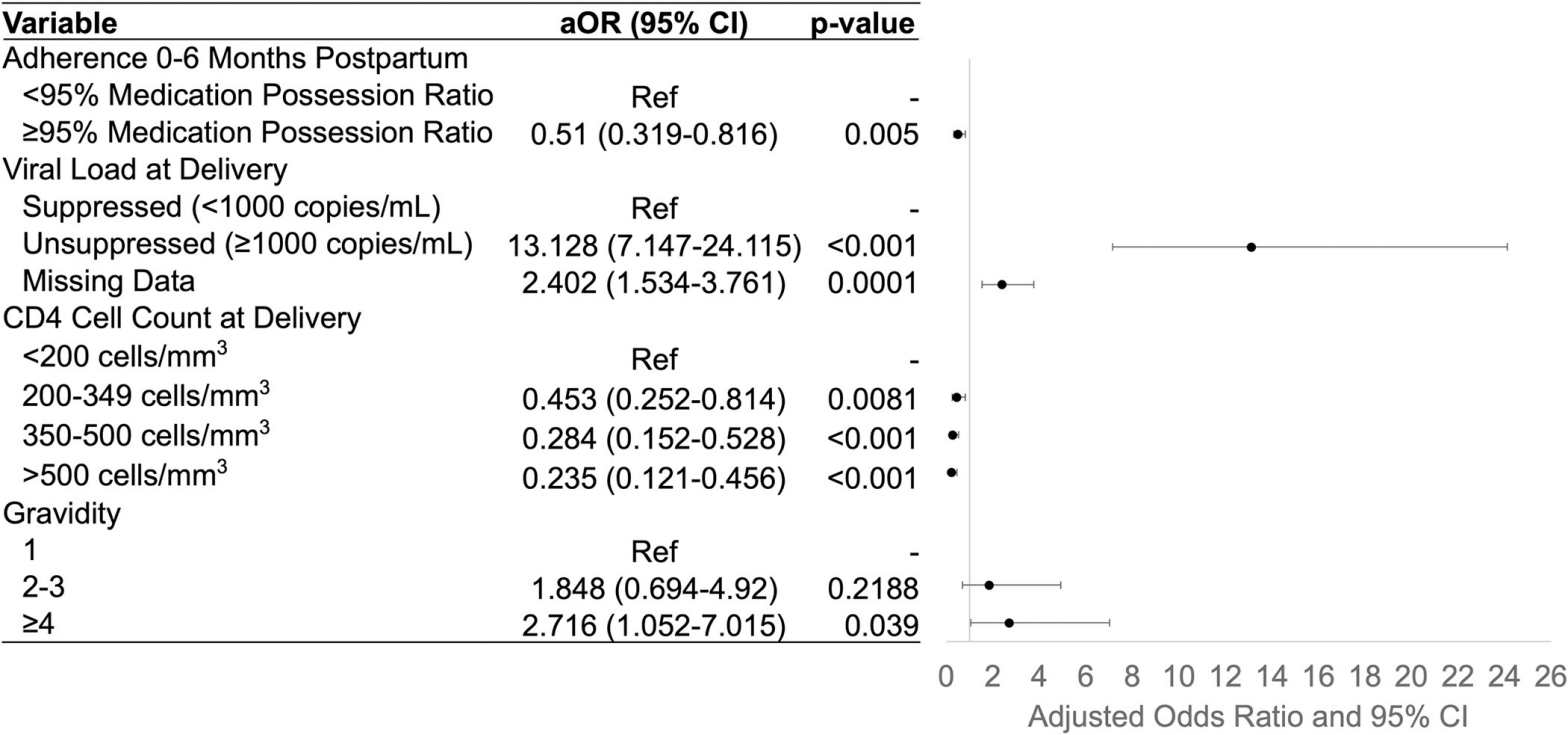
Risk factors for postpartum unsuppressed viral load. Final multiple logistic regression model shows significant risk factors for viral load non-suppression after delivery up to 24 months postpartum. Abbreviations: aOR, adjusted odds ratio; CI, confidence interval; ref, reference group.

The final multiple logistic regression model for unsuppressed viral load retained 866 patients (Fig 4). Having ≥95% ART adherence (aOR = 0.51, 95% CI 0.319–0.816) and higher CD4+ T-cell counts at delivery (200–349 cells/mm^3^, aOR = 0.453, 95%CI 0.252–0.814; 350–500 cells/mm^3^, aOR = 0.284, 95% CI 0.152–0.528; >500 cells/mm^3^, aOR = 0.235, 95% CI 0.121–0.456) were significantly protective against experiencing unsuppressed viral load postpartum. Significant risk factors for this adverse outcome were having an unsuppressed or missing viral load at delivery (aOR = 13.128, 95% CI 7.147–24.115 and aOR = 2.402, 95% CI 1.534–3.761, respectively) and having gravidity of ≥4 pregnancies (aOR = 2.716, 95% CI 1.052–7.015).

Our sensitivity analysis compared 422 women missing vs. 1024 women not missing postpartum viral load data (Table 4). Maternal age, marital status, time since HIV diagnosis, ART adherence, ART duration, viral load at delivery, CD4+ T-cell count at delivery, delivery year, and delivery type were potentially associated with missing data in bivariate analyses. The final multiple logistic regression model indicated that missing postpartum viral load data had significant inverse associations with having been on ART >8 years, ≥95% MPR, and delivery year after 2014.

**Table 4:** Sensitivity analysis–Patients with postpartum viral load data vs. patients missing data.

	Viral Load Recorded	Viral Load Missing	chi-square test	Multiple Logistic Regression	
	Number (%)	Number (%)	p-value	aOR^a^ (95% CI^b^)	p-value
**Demographic**					
Age at Delivery					
≤29 years	221 (63.0%)	130 (37.0%)	0.0005		
30–33 years	316 (71.7%)	125 (28.3%)			
34–36 years	223 (71.2%)	90 (28.8%)			
≥37 years	263 (77.4%)	77 (22.6%)			
Marital Status^c^					
Single/Separated/Divorced	351 (74.2%)	122 (25.8%)	0.0384		
Married	649 (68.9%)	293 (31.1%)			
Education Status^c^					
No Formal	74 (62.7%)	44 (37.3%)	0.0762		
Primary/Secondary	615 (70.4%)	259 (29.6%)			
Tertiary	309 (73.4%)	112 (26.6%)			
Occupation Status^c^					
Non-income Generating	454 (69.5%)	199 (30.5%)	0.2213		
Professional/Manager	248 (74.5%)	85 (25.5%)			
Labor/Service	294 (69.5%)	129 (30.5%)			
**Clinical HIV/ART** ^**d**^					
Previous ART Experience^c^					
ART Naive	884 (70.1%)	377 (29.9%)	0.1791		
ART Experienced	116 (75.3%)	38 (24.7%)			
Time since HIV Diagnosis					
Diagnosis during Pregnancy/Delivery	73 (57.9%)	53 (42.1%)	< .0001		
≤3 years Prepartum	120 (66.3%)	61 (33.7%)			
3.1–6 years Prepartum	235 (67.0%)	116 (33.0%)			
6.1–8 years Prepartum	212 (65.6%)	111 (34.4%)			
>8 years Prepartum	354 (84.3%)	66 (15.7%)			
Duration on ART prior to Delivery					
<4 years	285 (63.2%)	166 (36.8%)	< .0001	Ref^e^	Ref
4–8 years	380 (67.1%)	186 (32.9%)		0.88 (0.66–1.18)	0.3935
>8 years	307 (86.2%)	49 (13.8%)		0.47 (0.32–0.70)	0.0002
Drug Regimen at Delivery					
Regimens without a Protease Inhibitor	925 (70.7%)	383 (29.3%)	0.0624		
Regimens with a Protease Inhibitor	99 (78.6%)	27 (21.4%)			
Viral Load at Delivery					
Suppressed (<1000 copies/mL)	555 (72.9%)	206 (27.1%)	0.0030		
Unsuppressed (≥1000 copies/mL)	77 (58.3%)	55 (41.7%)			
Missing Data	392 (70.9%)	161 (29.1%)			
CD4 Cell Count at Delivery					
<200 cells/mm^3^	105 (61.0%)	67 (39.0%)	0.0015		
200–349 cells/mm^3^	261 (67.8%)	124 (32.2%)			
350–500 cells/mm^3^	296 (73.4%)	107 (26.6%)			
>500 cells/mm^3^	273 (75.8%)	87 (24.2%)			
Adherence 0–6 Months Postpartum					
<95% Medication Possession Ratio	210 (64.2%)	117 (35.8%)	0.0023	Ref	Ref
≥95% Medication Possession Ratio	814 (72.9%)	302 (27.1%)		0.40 (0.28–0.56)	<0.001
**Antenatal**					
Plurality					
1	886 (70.3%)	375 (29.7%)	0.8069^f^		
2–3	15 (75.0%)	5 (25.0%)			
Gravidity					
1	66 (76.7%)	20 (23.3%)	0.4031		
2–3	339 (70.0%)	145 (30.0%)			
≥4	548 (69.8%)	237 (30.2%)			
Previous Live Births					
0	134 (74.4%)	46 (25.6%)	0.4385		
1	195 (69.4%)	86 (30.6%)			
≥2	590 (69.9%)	254 (30.1%)			
Surviving Children					
0	155 (72.1%)	60 (27.9%)	0.7587		
1–2	478 (69.6%)	209 (30.4%)			
>2	284 (70.8%)	117 (29.2%)			
Previous Abortion					
0	529 (71.4%)	212 (28.6%)	0.2753		
≥1	355 (68.5%)	163 (31.5%)			
Trimester at First Antenatal Care Visit					
1st (≤12 weeks)	83 (79.0%)	22 (21.0%)	0.1409		
2nd (13–26 weeks)	652 (70.0%)	279 (30.0%)			
3rd (≥27 weeks)	278 (69.7%)	121 (30.3%)			
Total Antenatal Care Visits					
1–2	219 (70.9%)	90 (29.1%)	0.815		
3–4	412 (71.7%)	163 (28.3%)			
>4	393 (69.9%)	169 (30.1%)			
**Delivery**					
Year of Delivery					
2013–2014	339 (49.3%)	349 (50.7%)	< .0001	Ref	Ref
2015–2017	685 (90.4%)	73 (9.6%)		0.10 (0.07–0.13)	<0.001
Delivery Site					
Jos University Teaching Hospital	183 (69.6%)	80 (30.4%)	0.6024		
Other Clinic/Home/Road	833 (71.2%)	337 (28.8%)			
Delivery Type					
Vaginal/Assisted	699 (68.3%)	325 (31.7%)	0.0276		
Emergency/Elective C-Section	237 (74.8%)	80 (25.2%)			
Gestational Age at Delivery					
Pre-term (<37 weeks)	44 (73.3%)	16 (26.7%)	0.4943		
Full-term (≥37 weeks)	875 (69.2%)	390 (30.8%)			
Infant Birthweight					
Low (≤2.5 kg)	233 (66.2%)	119 (33.8%)	0.0927		
Normal/High (>2.5 kg)	688 (71.0%)	281 (29.0%)			
Infant Feed Method at Delivery					
Exclusive Breast Feeding	907 (71.6%)	360 (28.4%)	0.0611		
Breast Milk Substitute Supplement	95 (64.2%)	53 (35.8%)			

^a^aOR, adjusted odds ratio.

^b^CI, confidence interval.

^c^Denotes variables collected from the ART enrollment record, which was completed when the patient initiated ART in the APIN PEPFAR program. All other variables collected at antenatal booking or at delivery, as indicated.

^d^ART, antiretroviral therapy.

^e^ref, reference category.

^f^Fisher’s exact test p-values reported when contingency table observations were less than or equal to five.

## Discussion

To our knowledge, this retrospective analysis is the first to quantify both ART adherence and viral suppression up to 24 months postpartum and identify risk factors for LTFU and unsuppressed viral load in postpartum women with HIV in Nigeria.

In this study, 69.1% of patients had ≥95% MPR by 24 months postpartum–a proportion comparable to numbers observed in other sub-Saharan countries. Studies in Malawi using prescription pick-up data and South Africa and Zambia using self-reported adherence found 67%, 63.9%, and 70.5% of postpartum women with optimal adherence, respectively [22–24]. Definitions for optimal adherence varied slightly between 90% in the Malawi study and 100% in the South Africa and Zambia studies. We found a lower proportion of adherent postpartum women than a study in Abuja, Nigeria which found 82.9% adherent women using pill count [25].

Lower adherence rates in postpartum women compared with pregnant women with HIV have been documented [26]. We found a downward trend in ART adherence over the 24 months postpartum, from 79.0% of women with ≥95% MPR between months 0–6 to 69.1% of women with ≥95% MPR between months 18–24 postpartum. This postpartum decline again mimics the longitudinal trends of other sub-Saharan countries and identifies a crucial time period for intervention [10, 23, 24, 27].

The cumulative percentage of patients LTFU at 24 months postpartum in this study was 6.9%. This proportion is significantly lower than rates in other sub-Saharan countries such as Ethiopia and Malawi, where LTFU has ranged from 23%-24.5% [23, 24, 28]. The proportion LTFU in the general adult population with HIV in Nigeria has likewise been reported to be much higher, at 28% [29]. The focus on MTCT prevention at JUTH, through the APIN Public Health Initiatives, may have contributed to this improved retention among postpartum patients. Postpartum women with HIV may also be more motivated to continue ART (despite the difficulty in maintaining optimal adherence) during the first 24 months postpartum, while their infants are still being monitored for HIV infection. Exclusive breastfeeding was associated with viral suppression in bivariate analyses, suggesting a positive correlation with ART adherence, though not significant in the multiple regression, possibly due to collinearity with ART adherence which remained significant. Because our data were censored at 24 months, we could not assess outcomes afterward.

This study established risk factors for postpartum women becoming LTFU, previously unidentified in this patient population. Past studies have identified demographic determinants (i.e., younger age) and clinical determinants (i.e., viremia and missing CD4+ T-cell counts at delivery) as risk factors for pregnant women becoming LTFU after birth [30, 31]. Our study found the most significant risk factors for LTFU among postpartum patients were related to a patient’s engagement and amount of contact time with the HIV clinic and antenatal care before delivery. Having a more recent HIV diagnosis, fewer antenatal care visits, and a delivery outside of JUTH increased the risk of LTFU. Higher gravidity also increased the risk of LTFU; women with prior pregnancies likely have children to care for at home, and less time to care for their own health. Strategies for retaining the postpartum population in HIV care should, therefore, identify and engage these high-risk patients with enhanced adherence counseling during pregnancy and in the first postpartum year. While costly, studies indicate that patient tracing and repeated home visits are successful methods for reconnecting with patients after becoming LTFU [32, 33].

Our study found 85.8% viral suppression at month 12 and 88.7% viral suppression at month 24 postpartum among those with viral load results. These numbers fall short of the 95% UNAIDS target for viral suppression. In comparison, South African studies found 14.7%–14.8% postpartum viral non-suppression [34, 35]. Importantly, in our study, of the 1497 ART-eligible, only 50.0% had viral load results at month 12, and 57.3% had viral load results at month 24. Viral load monitoring is a challenge in many resource-limited settings. A South African study found only 12.6% of women had a viral load test by 9 months postpartum [34]. In our sensitivity analysis, patients missing postpartum viral load results were more likely to have shorter duration on ART and poorer adherence; therefore, our viral suppression rates in the postpartum population are likely overestimates. This may also explain why the proportion of patients with viral suppression did not decrease over the 24 months despite declining adherence. The 2018 Nigerian HIV/AIDS Indicator and Impact Survey found 77.1% viral suppression among adults on ART, which may be closer to what we might have observed in our study if viral load results were not missing [36].

Previous studies have identified younger age, shorter ART duration, and unsuppressed viral load at delivery as risk factors for unsuppressed viral load postpartum [12, 37, 38]. Our study similarly found unsuppressed viral load at delivery as a predictor of unsuppressed postpartum viral load; as unsuppressed viral load may indicate drug resistance, closer monitoring of these patients is needed. We additionally identified having poorer adherence, lower baseline CD4+ T-cell count, and more prior pregnancies as risk factors for unsuppressed postpartum viral load. Higher gravidity was a significant risk factor for both LTFU and unsuppressed viral load, and women with more children at home should be targeted for enhanced adherence counseling and supportive services.

While our study confirmed gaps in postpartum retention and adherence, solutions are not straightforward. Results of trials implementing phone calls or text message reminders to improve postpartum retention have been mixed [39–41]. Integrated care where mothers and infants are seen together at the PMTCT clinic postpartum has shown some promise. One study found 90% median ART adherence postpartum with 91% viral suppression in Uganda, where care was integrated, compared with 40% adherence postpartum with 57% viral suppression in South Africa, where women transferred to general ART services immediately after delivery [42]. A clinical trial found that integration of postpartum maternal and infant HIV care improved both retention and viral suppression at 12 months postpartum, but benefits did not continue after transfer to general ART services [43, 44]. Trials implementing ‘mentor mothers’ and community-based ‘adherence clubs’ have demonstrated improvements in viral suppression up to 24 months postpartum [45, 46].

While the large study population size and 24-month duration of individual postpartum follow-up strengthened this study, limitations remained. Our major limitation was missing data, with around 40% of viral load results missing at delivery and month 24, and 50% at month 12. Although viral load is the gold standard measure of ART effectiveness, it was not performed following the 12-monthly schedule. To accommodate for missing viral load data, missing baseline values were categorized separately and postpartum viral load time points were combined for the analyses, and a sensitivity analysis was performed to compare those with and without postpartum viral load results. Additionally, while MPR was used as a proxy measurement for ART adherence, without direct observation, it is possible that some patients picked up medication but did not adhere to their prescribed regimen. This study also exclusively focused on JUTH, a large urban tertiary hospital with an established HIV clinic since 2004, which may not be representative of Nigeria.

Finally, the impact of this study is limited by a lack of qualitative data. In addition to demographic and clinical barriers to ART retention and adherence like the ones we identified, significant individual (i.e., depression, understanding of ART), sociocultural (i.e., stigma, non-disclosure of HIV status), economic (i.e., financial resources, transportation), and structural (i.e., health worker attitudes) barriers persist for many people living with HIV in Nigeria and globally [25, 47–49]. Solutions to these long-standing barriers are elusive, as a Nigerian study that attempted a “continuous quality improvement” intervention found.[50] But so long as these barriers exist, any interventions may prove ultimately ineffective.

## Conclusions

As the risks for MTCT and adverse maternal health outcomes remain after birth, evaluation of retention, ART adherence, and viral suppression among postpartum mothers is critical. The cumulative percent of patients LTFU two years postpartum was lower for this Nigerian study population compared with postpartum patients in other sub-Saharan countries. As engagement with HIV and antenatal care decreases the risk of becoming LTFU, efforts to increase contact time among higher-risk patients should be initiated early in pregnancy. ART adherence among our postpartum population correlates with adherence rates in other sub-Saharan countries. The decline in adherence over the 24 months postpartum highlights the critical need for innovative adherence intervention strategies during this period. Viral suppression was considerably lower than the 95% UNAIDS target, and, importantly, the large percentage of missing viral load results was concerning. The causes of low viral load testing, whether policy-, funding-, and/or program-related must be addressed; point-of-care viral load monitoring should be considered.

For future studies, we recommend a longer follow-up time past 24 months to evaluate maternal retention, adherence, and viral load suppression after most infants have completed breastfeeding and HIV diagnostic testing. We also recommend including other urban and rural clinics in other regions for a more representative sample of postpartum women with HIV in Nigeria. Finally, we suggest surveys be administered throughout the MTCT prevention cascade to assess barriers to ART adherence and retention in care in this population as a start to understanding needs and considering interventions.
