# Supplementary material for: Antiretroviral therapy retention, adherence, and clinical outcomes among postpartum women with HIV in Nigeria
Source: PLoS One. 2024 Aug 7;19(8):e0302920. doi: 10.1371/journal.pone.0302920 (PMC11305526; doi:10.1371/journal.pone.0302920)
Supplement: Attachment — Submitted filename: Response to Reviewers.docx [file pone.0302920.s002.docx]

**Response to Reviewers**

**PONE-D-24-14899: Antiretroviral therapy retention, adherence, and clinical outcomes among postpartum women with HIV in Nigeria**

**July 1, 2024**

*Dear Editor and Reviewers,*

*We thank you for your review, feedback, and suggestions for improvement. We have read each point, and have addressed them below (in bold, italicized text) and/or in the manuscript (tracked and final), as appropriate. In line with PLOS’s Data Policy that promotes longevity and availability of the research data, we have added contact information for the APIN Public Health Initiatives Data Management Committee as a non-author institutional point of contact for data requests. We have also made minor edits for consistency of terminology and provided a breakdown of the unsuppressed baseline viral load category in Table 1, as suggested. Finally, we have used the PACE digital diagnostic tool to check our figures and have re-uploaded the corrected files. Kindly let us know if any of these or other items require further attention.*

*Sincerely,*

*Phyllis Kanki*

______________________________________________________________________________

**Journal requirements:**

When submitting your revision, we need you to address these additional requirements.

1. Please ensure that your manuscript meets PLOS ONE's style requirements, including those for file naming. The PLOS ONE style templates can be found at

https://journals.plos.org/plosone/s/file?id=wjVg/PLOSOne_formatting_sample_main_body.pdf and

https://journals.plos.org/plosone/s/file?id=ba62/PLOSOne_formatting_sample_title_authors_affiliations.pdf

***We have made some minor tracked revisions to ensure that our manuscript meets PLOS ONE’s style requirements to match the style templates.***

2. In this instance it seems there may be acceptable restrictions in place that prevent the public sharing of your minimal data. However, in line with our goal of ensuring long-term data availability to all interested researchers, PLOS’ Data Policy states that authors cannot be the sole named individuals responsible for ensuring data access (http://journals.plos.org/plosone/s/data-availability#loc-acceptable-data-sharing-methods).

Data requests to a non-author institutional point of contact, such as a data access or ethics committee, helps guarantee long term stability and availability of data. Providing interested researchers with a durable point of contact ensures data will be accessible even if an author changes email addresses, institutions, or becomes unavailable to answer requests.

Before we proceed with your manuscript, please also provide non-author contact information (phone/email/hyperlink) for a data access committee, ethics committee, or other institutional body to which data requests may be sent. If no institutional body is available to respond to requests for your minimal data, please consider if there any institutional representatives who did not collaborate in the study, and are not listed as authors on the manuscript, who would be able to hold the data and respond to external requests for data access? If so, please provide their contact information (i.e., email address). Please also provide details on how you will ensure persistent or long-term data storage and availability.

***We have added contact information for the APIN Public Health Initiatives Data Management Committee (dmc@apin.org.ng) as a non-author institutional point of contact for data requests. The APIN Public Health Initiatives Data Management Committee holds the dataset used for the analyses and will be able to respond to external requests for data access. APIN is a leading non-governmental organization active providing prevention, care and treatment services for diseases of public health significance and providing technical assistance to relevant government agencies in Nigeria since 2000. APIN owns and manages the participant-level clinical data used in this study. We have provided the cleaned dataset and codes used for our analyses to the APIN Data Management Committee, who will ensure long-term data storage and availability.***

3. Please review your reference list to ensure that it is complete and correct. If you have cited papers that have been retracted, please include the rationale for doing so in the manuscript text, or remove these references and replace them with relevant current references. Any changes to the reference list should be mentioned in the rebuttal letter that accompanies your revised manuscript. If you need to cite a retracted article, indicate the article’s retracted status in the References list and also include a citation and full reference for the retraction notice.

***We have no changes to the reference list.***

**Additional Editor Comments:**

Thank you very much for submitting your study to PLOSONE. This is an important study that evaluated ARVs retention, adherence and viral suppression among postpartum women in Nigeria. You raise an important justification for conducting the study, i.e. that there are very few studies that focus on this important period of pregnancy. This is a retrospective study involving a review of data previously collected for the APIN Public Health Initiatives HIV program at JUTH, an initiative that was supported by PEPFAR. A total of 1535 women living with HIV were reviewed ,1497 of whom were on ART. This study not only provide information on ART retention, adherence and viral suppression but also important information on the risk factors for loss of follow up during this important period. Your conclusion is valid and on point. There is a need for prospective studies that focus on providing solutions on how to improve ART retention among postpartum women including qualitative studies to further enlighten us on this topic. The design of the study is appropriate for the research questions and objectives. The same applies regarding the choice of statistical measures. Overall, the manuscript is scientifically sound and well written. The following suggestions are recommended to further improve the paper:

1) Consistency with terminology: Please consider using women living with HIV (WLHIV) or HIV Positive women and not both

***Thank you very much for your comments. For consistency, we have replaced the prior terminology to ‘women living with HIV,’ accordingly:***

***Page 3, Line 34: “ART coverage among pregnant and breastfeeding women living with HIV…”
Page 3, Line 38: “among pregnant women living with HIV…”
Page 4, line 59: “The study population included 1535 pregnant women living with HIV-1, …”***

2) If possible, consider creating sub-categories under the <1000 bin (e.g. <200 and 200 – 1000) because of the differences in the definition of viral suppression between different regions/countries as well as the differences between Nigeria and US/WHO guidelines. This is important for the international readership and other researchers who might want to replicate the study in their settings. Kindly also respond to the additional comments (attached)  from the reviewers.

***Thank you for this useful suggestion. The previous categories were chosen to align with the thresholds used in the Nigerian HIV care program and were maintained throughout the study analyses for consistency and data interpretation. In response to your observation and recommendation, we have added subcategories (<200cp/mL and 200-999cp/mL) to the baseline clinical indicator ‘Viral Load at Delivery’ in Table 1 to aid the international readership in understanding and contextualizing viral suppression in this study population. (A great majority of the previous ‘Suppressed (<1000 cp/mL)’ category were also <200 cp/mL.)***

Thank you once more for sharing your work


**Reviewers' comments:**

**Reviewer #1:** Thank you for the opportunity to review this well-written manuscript. It was a pleasure to read and highlights a very real problem in maternal and postpartum HIV care.

The authors conducted a retrospective review of data previously collected as part of the APIN Public Health
Initiatives HIV program at JUTH, supported by PEPFAR. In their study 1535 women living with HIV were reviewed and 1497 were on ART and thus eligible for further review in terms of viral suppression and loss to follow-up analysis. The authors showed that medication adherence (through medication possession ratio) declined from delivery through to 24 months postpartum and that close to 90% of women remained enrolled in care at 24 months post-delivery. Furthermore, the study adds to the literature by highlighting risk factors for loss to follow-up amongst postpartum women.

The authors raise a valid point in that further studies are needed to evaluate solutions for postpartum retention in care and the need for well-designed prospective studies to evaluate these variables. Furthermore, they highlight the need for qualitative studies in this field, something that is often shied away from.

I feel that the manuscript can be strengthened with two minor changes:

1) Please be consistent in your terminology – either “women living with HIV” or “HIV-positive women”

Page 3, Line 34: “ART coverage among pregnant and breastfeeding women with HIV…”
Page 3, Line 38: “among pregnant women with HIV…”
Page 4, line 59: “The study population included 1535 HIV-1-positive pregnant women”

***For consistency, we have replaced the prior terminology to ‘women living with HIV,’ accordingly:***

***Page 3, Line 34: “ART coverage among pregnant and breastfeeding women living with HIV…”
Page 3, Line 38: “among pregnant women living with HIV…”
Page 4, line 59: “The study population included 1535 pregnant women living with HIV-1, …”***

2) In table 1, would it be possible to include a subcategory under the <1000 bin (e.g. <200 and 200 – 1000)? Nigeria seems to have a more liberal definition of viral suppression (1000cp/ml) compared to the US and WHO guidelines of 200cp/ml, so this may aid international readers in understanding the burden of HIV in Nigeria and this data could be compared to other international studies in the future

***Thank you for this useful suggestion. The previous categories were chosen to align with the thresholds used in the Nigerian HIV care program and were maintained throughout the study analyses for consistency and data interpretation. In response to your observation and recommendation, we have added subcategories (<200cp/mL and 200-999cp/mL) to the baseline clinical indicator ‘Viral Load at Delivery’ in Table 1 to aid the international readership in understanding and contextualizing viral suppression in this study population. (A great majority of the previous ‘Suppressed (<1000 cp/mL)’ category were also <200 cp/mL.)***

Thank you again for the opportunity to review your manuscript.

**Reviewer #2:** The manuscript is well written and makes an important contribution to the literature postpartum viral suppression in women living with HIV.
The are some minor questions that the authors need to address.

Page 5, Line 69: Is the frequent (6-monthly) CD4 count monitoring standard for all adult patients?

***During the study period, the CD4 count monitoring schedule was every 6 months for all adult patients with HIV, as stated. We have added clarification in the text on Page 5, Lines 70-71. (Guidelines changed to less frequent monitoring in 2021, but after the study period.)***

Page 5, Line 70: Is viral load testing yearly even in those newly initiated on treatment, including pregnant women?

***In adults newly initiating treatment, viral load testing was recommended at 6 months, 12 months, and every 12 months thereafter. Pregnant women newly initiating treatment receive an additional test at initiation. We have clarified this in the text on Page 5, Lines 70-72 and 75–76.***

Page 12, Line 188: Are the values only referring to patients that didn’t have a suppressed viral load at delivery and postpartum?

***No, the mean viral load values refer to all patients that had any viral load result recorded. We have clarified this in the text on Page 12, Line 195.***

Page 24, Line 297: Could breastfeeding have also contributed to ART adherence in the first 24 months postpartum?

***Thank you for this question. Yes, it is possible that breastfeeding may have contributed to ART adherence postpartum. Women who breastfed exclusively were less likely to have unsuppressed viral load (133/907, 14.7%) than those who supplemented with breastmilk substitute (24/95, 25.3%) in bivariate analyses (p=0.0069) (Table 3). This indicates that exclusive breastfeeding was correlated with better adherence. Breastfeeding was not significant in the multiple regression model for viral suppression, possibly due to collinearity with ART adherence. We have added this suggestion in the text on Page 24-25, Lines 305-309.***

Page 29, Ref #6: Citation needs correction.

***Thank you for catching the error in Reference #6 which appears to be due to the built-in journal name in the reference management software we used. We have corrected the journal name from “AIDS Lond Engl” to its proper name which is just “AIDS” using our Reference Manager (untracked).***
